# Effect of positive end-expiratory pressure on additional passive ventilation generated by CPR compressions in a porcine model

**DOI:** 10.1186/s40635-021-00401-y

**Published:** 2021-07-26

**Authors:** Yosef Levenbrown, Md Jobayer Hossain, James P. Keith, Katlyn Burr, Anne Hesek, Thomas H. Shaffer

**Affiliations:** 1 Division of Pediatric Critical Care, Nemours/Alfred I. duPont Hospital for Children, 1600 Rockland Road, Wilmington, DE 19803 USA; 2 Department of Pediatrics, Sidney Kimmel Medical School of Thomas Jefferson University, Philadelphia, PA USA; 3 Nemours Biomedical Research, Wilmington, DE USA; 4 Department of Applied Economics and Statistics, University of Delaware, Newark, DE USA; 5 Department of Respiratory Care, Nemours/Alfred I. duPont Hospital for Children, Wilmington, DE USA; 6 Nemours Biomedical Research, Wilmington, DE USA; 7 Nemours Biomedical Research/Center for Pediatric Lung Research, Wilmington, DE USA; 8 Departments of Pediatrics and Physiology, Lewis Katz School of Medicine at Temple University, Philadelphia, PA USA

**Keywords:** Cardiac arrest, Resuscitation, Ventilation, Compressions, Tidal volume

## Abstract

**Background:**

Compressions given during cardiopulmonary resuscitation generate small, ineffective passive ventilations through oscillating waves. Positive end-expiratory pressure increases the volume of these passive ventilations; however, its effect on passive ventilation is unknown. Our objective was to determine if increasing positive end-expiratory pressure during cardiopulmonary resuscitation increases passive ventilation generated by compressions to a clinically significant point. This study was conducted on 13 Landrace-Yorkshire pigs. After inducing cardiac arrest with bupivacaine, cardiopulmonary resuscitation was performed with a LUCAS 3.1. During cardiopulmonary resuscitation, pigs were ventilated at a positive end-expiratory pressure of 0, 5, 10, 15, 20 cmH_2_O (randomly determined) for 9 min. Using the NM3 respiratory monitoring device, expired minute ventilation and volumetric capnography were measured. Arterial blood gas was obtained for each positive end-expiratory pressure level to compare the effects of positive end-expiratory pressure on carbon dioxide.

**Results:**

Increasing positive end-expiratory pressure from 0 to 20 cmH_2_O increased the mean (SEM) expired minute ventilation from 6.33 (0.04) to 7.33 (0.04) mL/min. With the 5-cmH_2_O incremental increases in positive end-expiratory pressure from 0 to 20 cmH_2_O, volumetric capnography increased from a mean (SEM) of 94.19 (0.78) to 115.18 (0.8) mL/min, except for 15 cmH_2_O, which showed greater carbon dioxide exhalation with volumetric capnography compared with 20 cmH_2_O. PCO_2_ declined significantly as positive end-expiratory pressure was increased from 0 to 20 cmH_2_O.

**Conclusions:**

When increasing positive end-expiratory pressure from 0 to 20, the contribution to overall ventilation from gas oscillations generated by the compressions became more significant, and may even lead to hypocapnia, especially when using positive end-expiratory pressures between 15 and 20.

## Background

Although a brief period of compression-only cardiopulmonary resuscitation (CPR) is currently recommended as an acceptable initial approach for an out-of-hospital cardiac arrest [1], studies demonstrate that ongoing CPR without ventilations will lead to worsening hypoxia and carbon dioxide retention resulting in a respiratory acidosis [2–4]. However, the optimal ventilation strategy during CPR is currently unknown [5]. The current recommendation to ventilate at a rate of one breath every 6 s when an advanced airway is in place is based on observational studies demonstrating that hyperventilation is common during CPR [6]. Cardiopulmonary resuscitation studies using a pig model further demonstrate that hyperventilation during CPR is associated with an increase in intrathoracic pressure, decrease in coronary and cerebral perfusion, and decreased rates of return of spontaneous circulation [7, 8].

Prior studies show that the changes in intrathoracic pressure generated by the chest compression and recoil of the thorax during CPR will result in some gas exchange. However, the volume of air mobilized through chest compressions alone is less than the physiologic dead space, and is therefore inadequate for ventilation and carbon dioxide removal, especially after the first few minutes of compressions [9–12]. Earlier studies show that airway closure occurs during compression and is responsible for minimizing the effect that these gas oscillations have on carbon dioxide clearance during CPR. More importantly, airway closure can potentially decrease the overall effectiveness of ventilation during CPR [13–15]. These limitations on carbon dioxide clearance can result in significant physiological consequences if suboptimal ventilation is performed during CPR, either due to suboptimal compression technique or due to the effect of chest compressions causing flow-reversal of tidal breaths during CPR [16]. Cardiopulmonary resuscitation models show that positive end-expiratory pressure (PEEP) can improve ventilation during CPR. As such, these studies speculate that the mechanism for improved ventilation with PEEP is possibly due to reversing airway closure that limits gas flow, thereby taking advantage of the oscillations of gas produced by the compression and decompression phases of CPR [14, 17]. However, the effect of different PEEP levels on improving ventilation generated by the compressions of CPR, as well as the optimal PEEP for ventilation remains undefined. In the current study, we sought to determine the effect of increasing PEEP from 0 to 20 cmH_2_O on ventilation, carbon dioxide elimination, and arterial carbon dioxide partial pressure during CPR.

## Methods

### Anesthesia and monitoring

This study was performed with the approval of the International Animal Care and Use Committee of Nemours/Alfred I. duPont Hospital for Children. This study was performed with Landrace-Yorkshire pigs weighing 35–38 kg (*n* = 13 pigs). The cohort of animals used in this study was part of a larger study, which analyzed the effect of PEEP on cardiac output during CPR [18]. After seeing trends in carbon dioxide shift with the various PEEP levels with the first animals in the study, data were collected to formally analyze effect of PEEP on carbon dioxide clearance for the last 13 animals in the study. The data reported here do not overlap in any way with the data presented previously.

The pigs received initial sedation with two intramuscular injections, 10 min apart, of 1 ml/kg of KAX, an anesthetic cocktail containing 23 mg/ml of ketamine, 0.58 mg/ml of acepromazine, and 0.8 mg/ml of xylazine. Following the initial sedation, a left carotid intra-arterial catheter and right internal jugular central venous catheter were placed using standard cut-down techniques. The pigs were then intubated via midline tracheostomy using a cuffed 7.0–7.5 endotracheal tube. The animals were connected to a ventilator (Servo-I, Getinge, Wayne, NJ, USA) and ventilated using volume control mode ventilation with a tidal volume of 8 mL/kg, a rate of 20 breaths per minute, PEEP of 5 cmH_2_O, and fraction of inspired oxygen of 1.0. The ventilator breath rate was subsequently adjusted to maintain pH in the 7.35–7.45 range. The volume of dead space in the ventilator tubing used in this study is 1,040 mL; however, automatic tubing compliance was utilized on the Servo-I ventilator to ensure that there was no gas lost in the circuit dead space, and that the dead space within the ventilator circuit was accounted for. The pigs then were given a bolus dose of 1 mg/kg of propofol and 15 mg/kg of ketamine and placed on infusions of 3 mg/kg/hour of propofol and 15 mg/kg/hour of ketamine. Bolus doses were repeated, and the continuous infusions were increased if the pig showed signs of pain, such as flinching, or showed a 10% increase in heart rate or blood pressure to a hoof pinch with a clamp. Following the sedation and the procedures, the pig was then given a 30-min stabilization period.

### Experimental protocol

Baseline (pre-cardiac arrest) arterial blood gas was obtained (Nova Biomedical, Stat Profile Prime Analyzers, Waltham, MA, USA). Bupivacaine, 6–9 mg/kg intravenously, was administered. This dose of bupivacaine causes an irreversible cardiac arrest in a porcine model [19, 20]. Cardiac arrest was confirmed by looking for asystole on the 3-lead cardiac monitor, as well as signs of no blood flow (loss of pulsatile arterial line tracing, loss of pulse oximeter tracing, and loss of end-tidal carbon dioxide tracing) for a full minute. After 1 min of cardiac arrest, a LUCAS 3.1 mechanical CPR compression device (Stryker, Kalamazoo, MI, USA) was applied to the pig, and compressions were started at a rate of 102 compressions per minute. The pigs continued to receive mechanical ventilation at the previously set rate, tidal volume, and FiO_2_ while receiving CPR, with the study protocol looking at the additional ventilation that occurs through the passive gas exchange generated by the compressions, superimposed on the background ventilations. In order to ensure optimum translation of experimental findings to clinical practice, the study utilized state-of-the-art pediatric intensive care instrumentation, monitors, and ventilators.

Each pig was then placed on a PEEP of 0, 5, 10, 15, and 20 cmH_2_O, with each PEEP level maintained for 9 min of CPR. The order of the PEEP levels that the pig was placed on was different for each study and was randomly determined by a lottery system that was performed in the beginning of each study. The lottery system entailed blindly picking out five folded up pieces of paper from an envelope, with each of the five pieces of paper having one of the five PEEP levels written on it. Effectiveness of ventilation for each PEEP level was measured by monitoring both the expired minute ventilation as well as volumetric capnography using the NM3 Respiratory Monitoring Device (Philips Healthcare, Amsterdam, Netherlands). Volumetric capnography allows measurement of the precise amount of carbon dioxide eliminated per breath by plotting the eliminated concentration of carbon dioxide versus the tidal volume of a single breath [21]. Measurements included in study analysis were the middle 5 min (minutes 3–7) of each PEEP level to avoid potential confounding effects of transitioning from one PEEP level to the next, as well as ensuring that the measurements used did not include the breaths delivered during the minute that the PEEP was changed. After 9 min on each PEEP level, arterial blood gas was obtained to monitor partial pressure of carbon dioxide (PaCO_2_).

### Statistical analysis

A mixed effects model with repeated measures analysis of variance was used to compare the mean of each expired minute ventilation (mL/min), volumetric capnography (VCO_2_ [mL/min]), and PCaO_2_ between PEEP levels assuming an autoregressive order [AR(1)] correlation structure among within-pig repeated measures. Tukey’s method of multiple comparison was used to compare the mean between each pair of PEEP levels. Model assumptions were checked before analysis. Time delay was utilized (delay from the first entry of the first PEEP level applied to each pig) as a repeated factor in the model to account for the heterogeneity of the allocation schedule. All tests were two-tailed with an overall level of significance of 0.05. Statistical software SAS, version 9.4 (IBM Corp., Armonk, NY, USA) was used for data analysis.

A mixed effects model with repeated measures analysis of variance was used to compare the mean outcomes for end-tidal carbon dioxide, pH, partial pressure of arterial carbon dioxide (PaCO_2_), bicarbonate, and lactate, respectively) over the different PEEP levels. A first-order autoregressive (AR1) correlation structure was used to account for the repeated measures of each pig for random sequence of PEEP levels over time. The least significant difference was used to compare pairwise mean differences between PEEP levels.

## Results

**Fig. 1:**
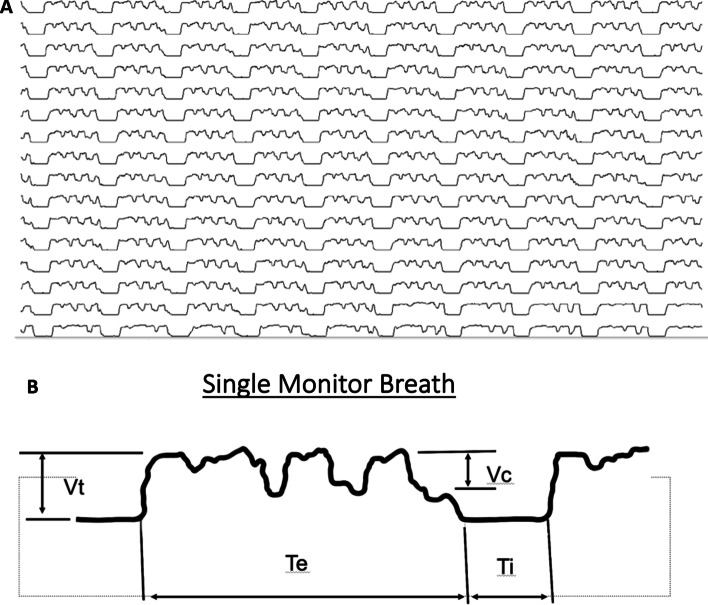
**A** Representative end-tidal carbon dioxide tracing obtained while performing cardiopulmonary resuscitation during this study demonstrating the oscillation of expired carbon dioxide superimposed on the typical end-tidal carbon dioxide tracing. The oscillations of air seen superimposed on the fixed breath were seen for all PEEP levels. **B** Identification of the components of a representative breath. Vt is exhaled tidal volume. Vc is volume of carbon dioxide moved during compressions. Te is exhalation time. Ti is inspiration time

Table 1 presents the estimated mean outcome for each PEEP level. As seen in Table 1, as PEEP was increased from 0 to 20, there was a significant increase in the mean (SEM) expired minute ventilation with 6.33 (0.04) L/min at PEEP 0 to 7.33 (0.04) L/min at PEEP 20. The values for minute ventilation include the set tidal volumes delivered by the ventilator plus the small passive ventilation breaths generated by the compressions. These passive ventilation breaths can clearly be seen superimposed on the ventilator-delivered breaths in Fig. 1. Given that the set minute ventilation delivered by the ventilator was constant throughout each study, the differences in minute ventilation seen with each of the PEEP levels were due to the differences in passive ventilation generated by the compressions. Volumetric capnography followed a similar pattern, increasing from a mean (SEM) of 94.19 (0.78) L/min to 115.18 (0.8) L/min as the PEEP was increased from 0 to 20. The only exceptions to this pattern were PEEP of 15 and 20 cmH_2_O, showing greater carbon dioxide exhalation with a PEEP of 15 compared with 20 cmH_2_O. There was a significant decline in PaCO_2_ as the PEEP was increased from 0 to 20 cmH_2_O, with PaCO_2_ decreasing from a mean (SEM) of 39.02 (2.59) mmHg at a PEEP of 0, to 27.67 (2.59) mmHg at a PEEP of 20.

**Table 1:** Estimated mean (SEM) outcome over positive end-expiratory levels

PEEP level	PEEP 0	PEEP 5	PEEP 10	PEEP 15	PEEP 20	*P* value
Variable	Mean (SEM)	Mean (SEM)	Mean (SEM)	Mean (SEM)	Mean (SEM)	
MVexp (L/min)	6.33 (0.04)	6.66 (0.04)	7.14 (0.04)	7.2 (0.03)	7.33 (0.04)	< 0.0001
VCO_2_ (L/min)	94.19 (0.78)	108.29 (0.81)	110.89 (0.83)	119.12 (0.77)	115.18 (0.8)	< 0.0001
PaCO_2_ (mmHg)	39.02 (2.59)	35.68 (2.59)	31.42 (2.59)	29.91 (2.59)	27.67 (2.59)	0.02

*MVexp* expired minute ventilation, *PaCO*_*2*_ partial pressure of carbon dioxide, *PEEP* positive end-expiratory pressure, *VCO*_*2*_ volumetric capnography

*P* value generated from mixed effects model to compare mean among PEEP levels

**Fig. 2:**
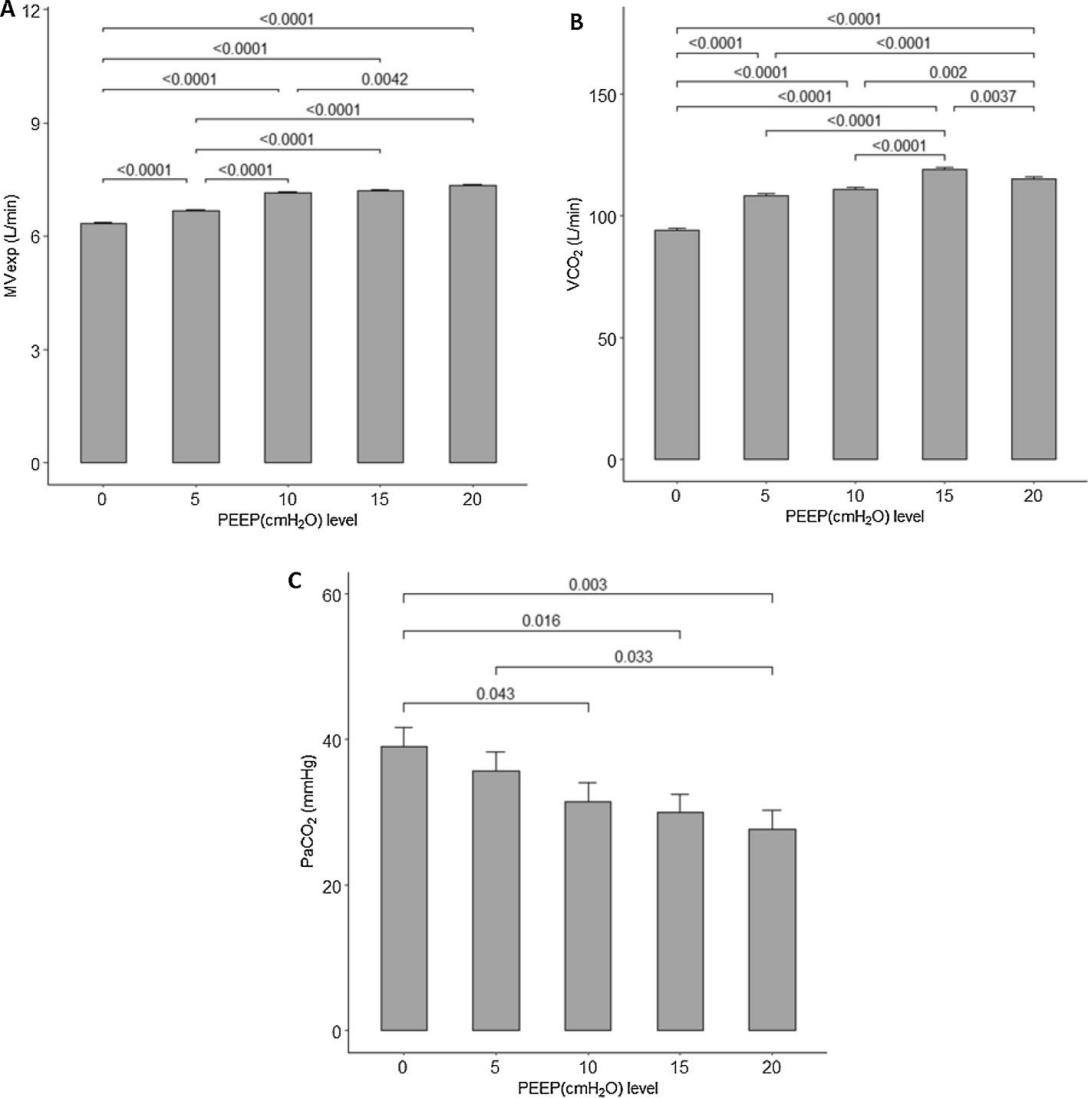
Bar graphs demonstrating the pairwise comparison of the expired minute ventilation (MVexp), (**A**); volumetric capnography (VCO_2_), (**B**); and arterial partial pressure of carbon dioxide (PaCO_2_), **C** for the different positive end-expiratory pressure (PEEP) levels

Table 2 as well as Fig. 2 shows the pairwise mean comparisons of the different PEEP levels for all three variables. In looking at the minute ventilation, volumetric capnography, and the PaCO_2_, the change in PEEP from 0 to 10 cmH_2_O generally had a greater effect than going from 10 to 20 cmH_2_O. In following the volumetric capnography, all increases in PEEP provided a significantly greater clearance of carbon dioxide, other than an increase from 5 to 10 cmH_2_O.

**Table 2:** Pairwise comparisons of outcomes between positive end-expiratory levels

Pairs of PEEP levels	MVexp_L/min		VCO_2_ L/min		PaCO_2_ mmHg	
	Mean diff (SE)	^$^*P* value	Mean diff (SE)	^$^*P* value	Mean diff (SE)	^$^*P* value
0, 5	− 0.33 (0.05)	< .0001	− 14.11 (1.12)	< .0001	3.33 (3.67)	0.367
0, 10	− 0.82 (0.05)	< .0001	− 16.7 (1.14)	< .0001	7.59 (3.67)	0.043
0, 15	− 0.88 (0.05)	< .0001	− 24.93 (1.09)	< .0001	9.11 (3.67)	0.016
0, 20	− 1 (0.05)	< .0001	− 21 (1.12)	< .0001	11.35 (3.67)	0.003
5, 10	− 0.49 (0.05)	< .0001	− 2.6 (1.16)	0.1656	4.26 (3.67)	0.250
5, 15	− 0.55 (0.05)	< .0001	− 10.83 (1.11)	< .0001	5.78 (3.67)	0.120
5, 20	− 0.67 (0.05)	< .0001	− 6.89 (1.14)	< .0001	8.02 (3.67)	0.033
10, 15	− 0.06 (0.05)	0.7795	− 8.23 (1.13)	< .0001	1.52 (3.67)	0.681
10, 20	− 0.18 (0.05)	0.0042	− 4.29 (1.16)	0.002	3.75 (3.67)	0.310
15, 20	− 0.12 (0.05)	0.0957	3.94 (1.11)	0.0037	2.24 (3.67)	0.544

*MVexp* expired minute ventilation, *PaCO*_*2*_ partial pressure of carbon dioxide, *PEEP* positive end-expiratory pressure, *VCO*_*2*_ volumetric capnography

^$^*P* value used to compare the mean difference between the corresponding pairs of PEEP levels. Tukey’s method was used to adjust the level of significance for multiple comparisons

As shown in Table 3, in comparing the mean outcomes for end-tidal carbon dioxide, pH, PaCO_2_, bicarbonate, and Lactate over the different PEEP levels, only pH (*p* = 0.05) and PaCO_2_ (*p* = 0.001) showed significant difference in overall mean comparisons among PEEP levels.

**Table 3:** Comparison of end-tidal carbon dioxide measurement (ETCO_2_), pH, arterial partial pressure of carbon dioxide (PaCO_2_), bicarbonate level (HCO_3_), and serum lactate for the different positive end-expiratory pressure (PEEP) levels applied during CPR

PEEP level	Estimated mean (SE)				
	ETCO_2_	pH	PaCO_2_	HCO_3_	Lactate
0	23.92 (1.56)	7.26 (0.03)	39.02 (2.71)	17.68 (1.17)	8.41 (0.82)
5	25 (1.56)	7.29 (0.03)	35.69 (2.71)	17.31 (1.17)	8.61 (0.82)
10	25 (1.56)	7.34 (0.03)	31.42 (2.71)	16.94 (1.17)	8.01 (0.82)
15	25.46 (1.56)	7.33 (0.03)	29.91 (2.71)	15.89 (1.17)	8.25 (0.82)
20	24.39 (1.56)	7.36 (0.03)	27.67 (2.71)	15.36 (1.18)	8.25 (0.82)
*P* value	0.803	0.05	0.001	0.301	0.906

*CPR* cardiopulmonary resuscitation, *SE* standard error

## Discussion

This current study was designed to determine whether escalating levels of PEEP can optimize carbon dioxide clearance by optimizing the gas flow and minute ventilation that occurs during chest compressions as well as mitigating the effect of airway closure on the delivered breaths from reaching the alveoli. The studies mentioned earlier demonstrate that PEEP can help facilitate alveolar ventilation during compressions; however, there are no studies looking at the optimal PEEP for alveolar ventilation resulting from the oscillations of gas that occur from the pressure changes due to the compressions during CPR. Thus, while keeping the ventilator rate the same for all PEEP levels, this study looked at expired minute ventilation and volumetric carbon dioxide as a measure of carbon dioxide elimination. This approach allowed us to evaluate the effect of PEEP on ventilation, both through improvements in ventilation that could be attributed to gas exchange due to oscillations of gas flow produced by the compressions, as well as maintenance of airway patency due to PEEP. The fact that compressions were causing these oscillations of gas flow, which enhanced carbon dioxide removal as PEEP was increased, was clearly indicated on the end-tidal carbon dioxide tracing, as seen in the representative tracing in Fig. 1, which is the end-tidal carbon dioxide tracing from one of the cases in this study. This figure clearly shows the oscillations from the compressions superimposed on top of the normal end-tidal carbon dioxide tracing. The impact that PEEP had on carbon dioxide clearance was determined by measuring both the exhaled minute ventilation as well as volumetric carbon dioxide measurements using PEEP of 0, 5, 10, 15, and 20 cmH_2_O. This study assumed that because ventilator settings (except PEEP) were constant and the quality of the compressions was consistent with the use of the LUCAS 3.1 for compressions, differences in ventilation between the PEEP levels would have been due to the oscillations of gas flow produced by the chest compressions, with the differences due to the effect of the different PEEP levels on these oscillations. In addition, PaCO_2_ was measured for each PEEP level to determine the overall effect that PEEP had on the measured carbon dioxide in the arterial blood gas.

The results of this study demonstrated that as PEEP was increased from 0 to 20 cmH_2_O, the minute ventilation as well as volumetric capnography increased significantly, with the exception of a PEEP of 15 cmH_2_O, during which the volumetric capnography was greater than for a PEEP of 20 cmH_2_O. In evaluating the effect that PEEP had on the arterial blood gas PaCO_2_, as the PEEP was increased from 0 to 20 cmH_2_O, there was a significant decrease in PaCO_2_.

When looking at the pairwise comparison of the different PEEP levels for all three variables, increasing the PEEP from 0 to 10 cmH_2_O had a more significant effect than increasing the PEEP from 10 to 20 cmH_2_O. However, it is important to note that, when ventilations were achieving adequate carbon dioxide clearance at lower PEEP levels, once PEEP was greater than 10 cmH_2_O, the PaCO_2_ was fairly low, in the 20s, all while keeping the compression and the set minute ventilation exactly the same. This finding would suggest, based on the results of this porcine model of CPR, that in a case where lung compliance is high and excessive PEEP is not needed for oxygenation as in our current model, it may be best to avoid higher PEEP levels during CPR, as higher PEEP levels in this current model of CPR even led to hyperventilation and hypocapnia. Avoiding hyperventilation is an important element of high-quality CPR. Excessive ventilation and associated hypocapnia results in respiratory alkalosis, inducing cerebral vasoconstriction further limiting oxygen delivery to the brain, which has already likely experienced significant hypoxia from the cardiac arrest no-flow state. Alkalosis also shifts the oxyhemoglobin curve to the left, possibly reducing oxygen off-loading to tissues [7, 22–24]. These findings, suggesting that the optimal PEEP during CPR should be less than 10 cmH_2_O when lung compliance is high, are in line with results from another study by our laboratory, which demonstrate that in terms of oxygen delivery during CPR, the optimal PEEP level was a PEEP of 5 cmH_2_O, which optimized both oxygenation and cardiac output to achieve the best oxygen delivery [18].

Whether the change in intrathoracic pressure generated by the compression and decompression of CPR can produce effective alveolar ventilation remains controversial. Chest compressions themselves can result in gas movement into the airway through the generation of subatmospheric pressures in the airway during the recoil phase of chest compressions as previously reported [10–12, 25]. However, the volume of air mobilized through chest compressions alone is less than the physiologic dead space, and is therefore inadequate for effective alveolar ventilation and carbon dioxide removal, most importantly after a few minutes of compressions [9–12]. Cordioli et al. demonstrate in five hospital cardiac arrest subjects that using zero PEEP, chest compressions produced extremely low ventilation that was flow limited, as depicted by end-tidal carbon dioxide tracings during CPR. The presumed cause for the extremely small, flow-limited ventilations during compressions was the reduction of lung volumes during chest compressions to below the end-expiratory thoracic volumes (functional residual capacity). This lung volume reduction led to closure of the distal airways, resulting in this flow limitation [9]. Airway closure during chest compressions can occur and limits ventilation, as initially shown by Safar and colleagues [26–30]. Their early studies indicate that the forces that oppose ventilation in cardiac arrest subjects are greater than those in subjects with preserved circulation. Also, Safar demonstrates that healthy volunteers undergoing chest compressions had a substantial amount of ventilation from the compressions. However, the gas exchange that is produced during chest compressions disappeared to almost nothing in cardiac arrest subjects. Possible etiologies for this ventilation limitation include narrowing of the bronchioles with lower lung volumes and closure of lung areas, presence of mucus, role of respiratory system elastic recoil with reduced pulmonary compliance, and reduced elasticity of the chest wall. This potential limitation in ventilation becomes more significant as the duration of CPR increases [9, 13, 14]. These findings are consistent with prior studies demonstrating that when end-expiratory lung volumes fall below the closing capacity of the lungs, airway collapse results [15].

Additionally, CPR leads to significant alveolar derecruitment and a decrease in pulmonary compliance [9, 13, 31]. When the lung volume decreases below the closing capacity, the small distal airways are likely to collapse, decreasing lung compliance. Investigators speculate that intrathoracic airway closure eliminates the transmission of negative alveolar pressure generated during chest recoil. Thus, negative alveolar pressure is not facilitating airway opening and therefore does not generate any inspiratory flow despite a significant pressure gradient [17].

During compression-only CPR, sustained levels of end-tidal carbon dioxide are present in most subjects. However, despite the fact that the tidal volumes generated from the compression are approximately only 27% of the total dead space, alveolar gas exchange is occurring during compressions [10]. The question thus becomes can anything be done to augment the alveolar ventilation attributed to the compressions during CPR? If for some reason ventilation is being performed inadequately during CPR, augmenting the alveolar gas exchange due to the change in pressure from the compression and decompression phase of CPR can potentially assist in the ventilation effort. Reasons why ventilation performed during CPR may be inadequate could be related to technique or may be intrinsic to the effect that the compressions have on the intrathoracic pressures. This effect could include airway closure or flow reversal of manual ventilation pulses that occur when ventilator-delivered breaths interact with chest compressions [16].

When the airway pressure is adequate to overcome the closing pressure, airway patency permits transmission of thoracic pressure at the airway opening and may allow some ventilation from chest compression [14]. Studies performed by the CAVIAR (Cardiac Arrest and Ventilation International Association for Research) group demonstrate that in a cadaver model and a bench model of CPR, as well as, in a clinical study analyzing capnograms of intubated subjects receiving CPR, the airway closure that results in the flow limitations during compressions was mitigated by the use of PEEP of up to 10 cmH_2_O. In this regard, PEEP allowed some degree of ventilation to occur with the oscillations of airflow generated by the change in intrathoracic pressure associated with chest compressions and decompressions [13, 14]. Their models demonstrate that airway closure, which occurred when PEEP was not utilized during CPR, was associated with an absence of carbon dioxide oscillations from chest compressions, which can significantly impair carbon dioxide clearance. Partial airway closure can also affect ventilation and carbon dioxide clearance. Opening index, which was their bedside method of estimating airway patency of 20% to 70%, was still associated with low minute ventilation [14]. Thus, small amounts of PEEP (< 10 cmH_2_O) were able to maintain the airway patency, allowing the pressure gradient from the compression/recoil phases of CPR to reach the upper airway, improving effective alveolar ventilations caused by the compressions [14, 17].

Thus, although we cannot prove from our study results that the increased carbon dioxide clearance seen with the application of PEEP is due to reversing distal airway collapse during CPR, our results (enhanced clearance of carbon dioxide with the use of PEEP during CPR) are consistent with other studies. As suggested in multiple other studies, the proposed mechanism for this effect is reversal of distal airway collapse during the compression phase of CPR [9, 26–30]. This current study adds to the prior studies in the demonstration and quantitative effects of incremental increases in PEEP on carbon dioxide clearance during CPR. As noted by our findings, airway collapse was not apparent, presumably given the fact that proposed distal airway collapse during CPR would not be readily apparent on the end-tidal tracing.

In addition to the impact this improved ventilation may have on resuscitation attempts, if carbon dioxide clearance is falsely decreased due to airway closure, end-tidal carbon dioxide (a surrogate marker for metabolic activity during CPR) may be falsely decreased. Because observational studies have shown that an end-tidal carbon dioxide less than 10 mmHg for more than 20 min during CPR can predict mortality with a positive and negative predictive value of 100% [32], this effect of airway closure may lead unfortunately to premature termination of resuscitation efforts based on a misleading low end-tidal carbon dioxide. Thus, it is important to utilize an appropriate amount of PEEP to maintain airway patency during CPR so the end-tidal carbon dioxide accurately reflects metabolic activity during CPR, which, when low, can be used as a meaningful predictor of outcome. Furthermore, this study also highlights a potential benefit of ventilating a patient through an endotracheal tube during CPR, rather than utilizing a supraglottic airway, as the supraglottic airway devices are less capable of maintaining PEEP compared with an endotracheal tube.

Finally, as in most model systems, this study has a number of limitations. Firstly, most CPR studies evaluating the physiology of CPR are done on animals or other non-human models. Although the porcine model is commonly used as a model for cardiac arrest because their physiology approximates that of humans [19], clearly there are differences in cardiovascular physiology between humans and pigs, such as different thorax geometry [14] that make it an imperfect model for CPR physiology in humans. However, it is not possible to obtain the magnitude of controlled data during CPR performed on humans during an actual cardiac arrest. Therefore, the only way to better understand the physiology of CPR is through animal, cadaver, or mechanical models. Secondly, the outcomes in this study are meant to evaluate cardiovascular physiological parameters during CPR with the goal of optimizing gas exchange during human CPR. For this reason, the laboratory experimentation was performed with state-of-the-art intensive care clinical instruments, monitors, and ventilators for optimum translation of outcomes to clinical practice. However, there is no evidence that following these conclusions will directly lead to a better outcome in human cardiac arrest subjects, since cardiac subjects may have additional confounding health issues that the studied pigs, who were all certified as healthy, did not. Further studies need to be performed to demonstrate that these results could result in improved outcomes during CPR. Thirdly, after initiating cardiac arrest and during the 60 s prior to initiation of chest compression, the pigs were ventilated using a PEEP of 5 cmH_2_O to avoid alveolar collapse prior to the implementation of the study protocol. This practice differs from that of typical cardiac arrest subjects who are not receiving PEEP when they go into cardiac arrest. We feel that these limitations do not detract from the results of this study, as the goal of the study was to look at the impact PEEP has on ventilation, carbon dioxide elimination, and arterial carbon dioxide partial pressure during CPR, which this study certainly does. In addition, the conclusions reached in this study were based on the cumulative effect of the different PEEP levels over a 5-min period on the gas exchange occurring from the chest compressions that was superimposed on the ventilator-delivered breaths. Due to the rapid pace of and small volumes of the gas movement that were generated by the chest compressions, the tidal volumes of these individual breaths were unable to be measured directly. Finally, we do acknowledge that PaCO_2_ potentially can be lowered by a decrease in cardiac output that is seen as PEEP is increased as opposed to reflecting an increase in ventilation, as presented in this study. However, as seen in Table 1, the decrease in PaCO_2_ parallels the increase in minute ventilation seen as PEEP is increased. Also, although it may be true that a decrease in cardiac output can result in decreased PaCO_2_, we feel that the value of including the volumetric capnography in this study is precisely that, to demonstrate that the decline in PaCO_2_ is related to the increased minute ventilation as opposed to a decrease in cardiac output. Given that volumetric capnography is a reflection of minute ventilation and exhaled carbon dioxide, if the decrease in PaCO_2_ was due to a decrease in cardiac output, one would expect the volumetric capnography numbers to decrease as PEEP was increased. Instead, we see that, other than for PEEP of 15 and 20 cmH_2_O, which show a slightly higher number for the volumetric capnography for PEEP of 15 compared with 20 cmH_2_O, the volumetric capnography does not follow the trend of the minute ventilation, increasing as PEEP is increased. (Perhaps the higher volumetric capnography value for PEEP of 15 compared with 20 cmH_2_O can be explained by the greater decrease in cardiac output seen at a PEEP of 20 compared with 15 cmH_2_O, as was demonstrated previously by this group [18].

## Conclusions

It is a well-known phenomenon that the intrathoracic pressure changes that occur during the compression and decompression phases of CPR produce a tidal volume that generally does not contribute significantly to gas exchange during CPR because these tidal volumes are less than the anatomical dead space. This study demonstrated that as PEEP is increased from 0 to 20 cmH_2_O, the contribution to overall ventilation from these gas oscillations generated by the compressions became more significant, and could potentially lead to a hypocapnia, especially when PEEP between 15 and 20 cmH_2_O was used in lungs with normal compliance.

## Data Availability

The datasets generated and/or analyzed during the current study are available from the corresponding author on reasonable request.

## Abbreviations

CPR: Cardiopulmonary resuscitation
PEEP: Positive end-expiratory pressure
PaCO_2_: Partial pressure of carbon dioxide
VCO_2_: Capnography
